# Cave morphology, microclimate and abundance of five cave predators from the Monte Albo (Sardinia, Italy)

**DOI:** 10.3897/BDJ.8.e48623

**Published:** 2020-02-03

**Authors:** Enrico Lunghi, Claudia Corti, Manuela Mulargia, Yahui Zhao, Raoul Manenti, Gentile Francesco Ficetola, Michael Veith

**Affiliations:** 1 Key Laboratory of the Zoological Systematics and Evolution, Institute of Zoology, Chinese Academy of Sciences, Beijing, China Key Laboratory of the Zoological Systematics and Evolution, Institute of Zoology, Chinese Academy of Sciences Beijing China; 2 Museo di Storia Naturale dell'Università degli Studi di Firenze, "La Specola", Firenze, Italy Museo di Storia Naturale dell'Università degli Studi di Firenze, "La Specola" Firenze Italy; 3 Universität Trier Fachbereich VI Raum-und Umweltwissenschaften Biogeographie, Trier, Germany Universität Trier Fachbereich VI Raum-und Umweltwissenschaften Biogeographie Trier Germany; 4 CEAS Santa Lucia, Siniscola, Italy CEAS Santa Lucia Siniscola Italy; 5 Department of Environmental Sciences and Policy, Università degli Studi di Milano, Milano, Italy Department of Environmental Sciences and Policy, Università degli Studi di Milano Milano Italy; 6 Université Grenoble Alpes, CNRS,, Grenoble, France Université Grenoble Alpes, CNRS, Grenoble France; 7 LECA, Laboratoire d’Ecologie Alpine, Grenoble, France LECA, Laboratoire d’Ecologie Alpine Grenoble France; 8 Université Savoie Mont Blanc, Annecy, France Université Savoie Mont Blanc Annecy France

**Keywords:** Dataset, standardised data collection, cave biology, troglophiles, salamander, spider, snail, monitoring, endangered species

## Abstract

**Background:**

Systematic data collection on species and their exploited environments is of key importance for conservation studies. Within the less-known environments, the subterranean ones are neither easy to be studied, nor to be explored. Subterranean environments house a wide number of specialised organisms, many of which show high sensitivity to habitat alteration. Despite the undeniable importance to monitor the status of the subterranean biodiversity, standardised methodologies to record biotic and abiotic data in these environments are still not fully adopted, impeding therefore the creation of comparable datasets useful for monitoring the ecological condition in the subterranean environments and for conservation assessment of related species.

**New information:**

In this work we describe a methodology allowing the collection of standardised abiotic and biotic data in subterranean environments. To show this, we created a large dataset including information on environmental features (morphology and microclimate) and abundance of five predators (one salamander, three spiders and one snail) occurring in seven caves of the Monte Albo (Sardinia, Italy), an important biodiversity hotspot. We performed 77 surveys on 5,748 m^2^ of subterranean environments througout a year, recording 1,695 observations of the five cave predators. The fine-scale data collection adopted in our methodology allowed us to record detailed information related to both morphology and microclimate of the cave inner environment. Furthermore, this method allows us to account for species-imperfect detection when recording presence/abundance data.

## Introduction

Subterranean environments represent peculiar habitats with an extraordinary biodiversity, including species with unique adaptive traits (Culver and Pipan 2015, Ficetola et al. 2019, Mammola 2019). Subterranean environments, ranging from small and narrow crevices to great and deep natural (or artificial) caves, are semi-closed systems with, at least, one connection with the surface (Culver and Pipan 2019). Thus, external climate influences the subterranean microclimate, in particular in its shallow parts (Badino 2004, Badino 2010, Culver and Pipan 2014). Subterranean environments are generally divided into different ecological zones according to the intensity of external influence (Culver and Pipan 2019); this differentiation of microhabitats promotes local biodiversity (Campbell Grant et al. 2007, Culver and Pipan 2010). The entrance and twilight zones of the cave are the parts most influenced by the external climatic conditions, while, far from the surface, such influence becomes weaker and the microclimate is more stable (Lunghi et al. 2015, Culver and Pipan 2019).

Subterranean environments are inhabited by peculiar animal communities (Culver and White 2005, Romero 2009, Romero 2011). Species living in subterranean environments are subdivided into three macro-categories according to their degree of adaptation to the subterranean environment (Pavan 1944, Howarth and Moldovan 2018). The trogloxenes are species showing little or no adaptation and are often an accidental presence in subterranean environments. Troglophiles are species that can show some adaptations to cave life and are able to spend at least part of their life in subterranean habitats. Troglobites are obligate cave species showing the highest adaptation to these particular environments. Species commonly thought to visit caves accidentally were usually considered of least importance for subterranean habitats (Sket 2008, Trajano and de Carvalho 2017); however, recent studies showed that some of them are actually selecting areas with specific conditions (Lunghi et al. 2014, [Bibr B5444560][Bibr B5444415]) and, in some cases, they can hold a key role in supplying organic matter to the entire ecosystem (Barzaghi et al. 2017, Culver and Pipan 2019). Most of the cave-related species are usually very sensitive to habitat alterations, a condition promoted by the high stability of the subterranean microclimate (Rizzo et al. 2015, Mammola et al. 2019a). The adaptation to the narrow range of environmental conditions present in these particular habitats, promotes a reduced home range and dispersal ability in subterranean organisms, often resulting in high rates of endemism (Manenti et al. 2018, Culver and Pipan 2019). This feature contributes to increasing the risk of extinction of cave animals (Williams et al. 2009, Lunghi et al. 2019) and thus, particular attention and updated information are needed to protect the subterranean environments and their inhabiting biodiversity (Ficetola et al. 2019, Mammola et al. 2019b).

**Figure 1.**
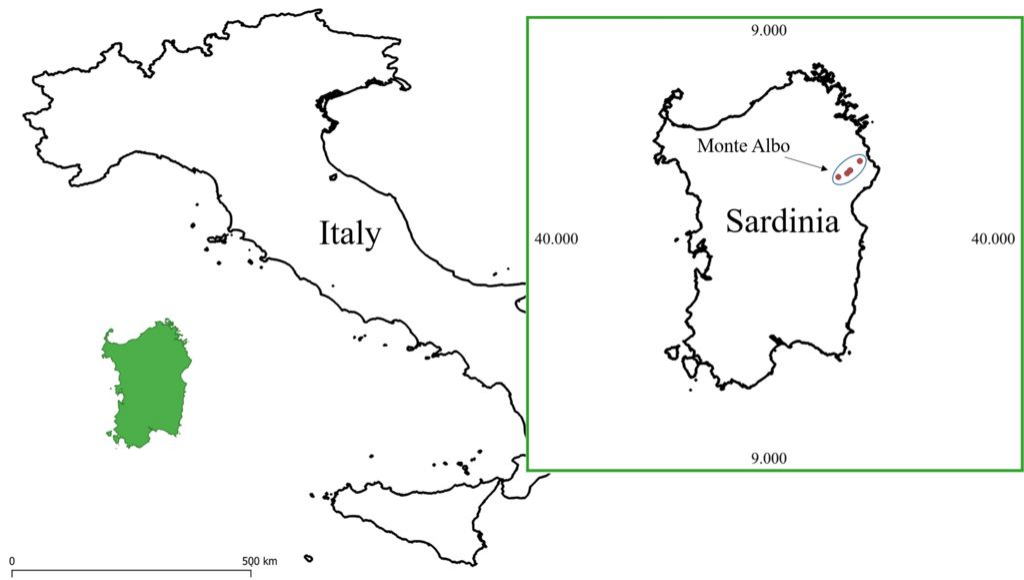
Map of the study area showing the location of the Monte Albo and the seven monitored caves (pink circles). Detailed representation of the map is avoided to increase species protection (Lunghi et al. 2019).

Considering the complexity of exploring subterranean habitats (Zagmajster et al. 2010), information on these environments is generally limited to a small group of specialists and have coarse scales. For example, the information related to the cave inner morphology usually describes the overall shape of the subterranean space and it is often published in local speleological bulletins or cadasters. Moreover, studies on cave microclimate rarely focus on fine-scale data collection (Cigna 2002), which is the most reliable information to detect potential alterations of the environment due to human activities (Langer 2001, Marín et al. 2012, Mammola et al. 2019b), as well as to assess the microclimatic conditions actually experienced by species characterised by limited home ranges (Campbell Grant et al. 2007, Lunghi et al. 2017, Ficetola et al. 2018a, Mammola et al. 2018). Today, we observe a growing interest in the ecology and biodiversity of subterranean environments, as testified by the increasing amount of available literature (Romero 2009, Moldovan et al. 2018, Mammola 2019, White et al. 2019) and related datasets (Lunghi et al. 2018c, Lunghi et al. 2019b, Mammola et al. 2019c); however, a standardised methodology to record data in these environments is still not widely adopted, therefore impeding the creation of comparable datasets (Wynne et al. 2019). Here we provide a detailed description of a methodology useful to record fine-scale standardised biotic and abiotic data in subterranean environments. Using this approach, we produced a large dataset containing fine-scale data describing the morphology and microclimate of the inner environment from seven caves located in the Monte Albo massif (Sardinia, Italy) (Fig. 1). Furthermore, this approach also allowed us to record data on species presence and abundance, information of key importance for assessing species conservation status (Ficetola et al. 2017, Ficetola et al. 2018b).

## General description

### Purpose

The aim of this paper is to describe a standardised method to record fine-scale ecological and biological data in subterranean environments. To prove this, we here provide a large dataset recorded using the proposed approach (Suppl. material 1). The dataset includes morphological and microclimatic features from seven caves located in the Monte Albo massif (Sardinia, Italy). Furthermore, the dataset includes the abundance of five cave predators (Fig. 2): *Hydromantes flavus* Stefani, 1969 (Urodela: Plethodontidae), *Meta bourneti* (Roberts, 1995) (Araneae: Tetragnathidae), *Metellina merianae* (Scopoli, 1763) (Araneae: Tetragnathidae), *Tegenaria* sp. Latreille, 1804 (Araneae: Agelenidae), *Oxychilus oppressus* (Shuttleworth, 1877) (Gastropoda: Oxychilidae). Data here are integrated with those published in Lunghi (2018).

### Additional information

Recommendations to adopt this survey method:

- This methodology was designed to monitor the endangered European cave salamanders of the genus *Hydromantes* (Ficetola et al. 2012, Lunghi et al. 2014, Lunghi et al. 2015), therefore the cave inner environment was divided according to the home range of these salamanders in subterranean environment (~3 m^2^; Lanza et al. 2006). Although this methodology is also useful to monitor a wide number of taxa, including frogs, toads, crickets, spiders and slugs/snails (Lunghi et al. 2017, Lunghi 2018, Lunghi et al. 2018a), we recommend to adapt the size of cave sectors based on the biology of the target species;
- We here provide recommendation only on the length of cave sectors (i.e. 3 linear metres long), but not on the width. In our case, the inner cave environments did not reach a significant width (cave sector width always < 15 m and generally < 3 m); we therefore suggest to consider horizontally subdividing the cave sectors when the area is particularly wide;
- The species monitored here are generally found on cave walls and on the ground; if researchers need to also include the ceiling in their survey, we suggest to increase the searching time to 10 min per cave sector;
- The Visual Encounter Survey adopted here allows the collection of data only on species easily detectable by sight, while for those particularly secretive, alternative approaches are needed (Wynne et al. 2019);
- The easily detectability of our target species and the relatively small monitored area, allowed us to collect reliable data within the defined monitoring time; however, the time and/or cave sector size may be changed according to specific requirements.

## Sampling methods

### Study extent

We monitored 7 caves located in different areas of the Monte Albo massif (Fig. 1); surveys were performed at least twice per season (with a gap of 1 to 7 days between the two seasonal surveys), starting from Autumn 2015 to Summer 2016. We performed a total of 77 surveys (average ± SD per cave; 11.43 ± 4.39) throughout a year (Autumn, *N* = 14; Winter, *N* = 14; Spring, *N* = 35; Summer, *N* = 14). Each pair of surveys was performed by day (9 a.m. – 6 p.m.) and when similar meteorological conditions occurred (i.e. sunny days with similar air temperature and humidity). Each cave was divided into 3 longitudinal linear metres sections (hereafter, sectors) in order to collect fine-scale data of the inner environment (Ficetola et al. 2012, Lunghi et al. 2014). Overall, we monitored 179 cave sectors for a total of 5,748.35 m^2^ (ceilings were not considered as they were usually too high). For each cave, we measured the maximum height and width of the main entrance, while, at the end of each cave sector, we recorded: maximum height, maximum width and average wall irregularity (see Abiotic data collection – Morphology). During each season, at 5-10 m from the cave entrance, we recorded the external air temperature and humidity. At the end of each cave sector, we seasonally recorded the following data: average air temperature, average air humidity, maximum and minimum illuminance (see Abiotic data collection – Microclimate). Within each sector, we recorded the abundance of five predator species (see Species data collection), providing a total of 1,695 observations: *H. italicus*, *N* = 831; *M. bourneti*, *N* = 182; *M. merianae*, *N* = 351; *Tegenaria* sp., *N* = 151; *O. oppressus*, *N* = 180 (Fig. 2).

### Sampling description

**Abiotic data collection**

*Morphology*

Caves were explored entirely or up to the point reachable without speleological equipment. Using a laser meter (Anself RZE-70, accuracy 2 mm), we recorded the maximum height and width of the cave entrance (i.e. the main connection with the external environment). Using a tape meter, the cave environment was divided into 3-metre cave sectors. At the end of each cave sector, we recorded the maximum height and width using a laser meter. At the same point, we estimated the average maximum wall irregularity (i.e. presence of wall protuberances). To estimate wall irregularity, we used a string of one metre length, flattened vertically against the most irregular part of each cave wall (left and right), at a height ranging from 0.5 to 2 m; a tape meter was then used to measure the linear distance between the two extremities of the string (Ficetola et al. 2012, Lunghi et al. 2014). We then merged the data and obtained the average cave sector maximum wall irregularity.

*Microclimate*

During each season, air temperature and humidity were recorded in the external surroundings of each cave, in a shaded area 5-10 m from the entrance, using a Lafayette TDP92 thermo-hygrometer (accuracy: 0.1°C and 0.1%). At the end of each cave sector, the average air temperature and humidity were estimated by averaging data recorded at ground level and at 2.5 m height (or at the ceiling if sector height was lower). Microclimatic data were recorded paying attention to limit researcher influence (Lopes Ferreira et al. 2015). At the end of each sector, the maximum and minimum incident light was measured using a Velleman DVM1300 light meter (minimum recordable light: 0.1 lx).

**Species data collection**

Data on species occurrence and on the number of detected individuals were obtained using the Visual Encounter Survey (i.e. the surveyor visually inspected the whole cave sector without disturbing species) (Crump and Scott 1994). Within each cave sector, the surveyor dedicated 7.5 minutes in assessing the presence/abundance of the target species (Lunghi et al. 2015, Lunghi et al. 2017, Lunghi 2018); the use of a fixed time within each cave sector allows the surveyor to limit potential effects of imperfect species detection (Banks-Leite et al. 2014). When at least one individual was observed within the cave sector, the species was considered present; when no individuals were observed, the species was considered absent. For the three spiders, the number of cocoons observed per sector was also recorded.

### Quality control

A dataset is provided to be readily used with R statistical software.

Several scientific studies support the reliability of the monitoring methodology proposed here (Ficetola et al. 2012, Ficetola et al. 2018a, Lunghi et al. 2014, Lunghi et al. 2015, Lunghi et al. 2017, Lunghi 2018, Lunghi et al. 2018a, Lunghi et al. 2018b).

The standardised methodology adpoted here and its overall repeatability, allowed the collection of comparable data from different environments during multiple time series (e.g. seasons or years). This makes it possible to identify potential changes of the local environmental conditions, giving the chance to promptly plan habitat conservation actions (Langer 2001, Marín et al. 2012, Mammola et al. 2019b).

Species can be overlooked, especially the small-sized ones: a lack of observation does not mean a true absence (MacKenzie et al. 2006). Adopting a standardised monitoring method allows the limitation of biases due to imperfect species detection (Banks-Leite et al. 2014, Lunghi 2018); in our case, we standardised the effort (time/sector) dedicated in searching of species.

Sites were surveyed twice per season with a maximum gap ≤ 7 days, allowing us to meet prerequisites for population closure and to limit variation of climate conditions, which can, in turn, affect individuals’ activity (MacKenzie et al. 2006, Lunghi et al. 2015). The two seasonal presence/absence data collected within this short time allowed us to statistically estimate the probability to detect the target species. The seasonal pair of surveys performed here allowed us to estimate species detection probability for each season, providing, therefore, important information on species occurrence throughout the year.

Abundance data collected in a relatively short time allows us to estimate population size, thus providing the fundamental information to perform species conservation assessments (Ficetola et al. 2017, Ficetola et al. 2018b). The multiple counts of individuals can be analysed with the *N*-mixture models to estimate the population size; the higher the number of counts, the more precise the estimation will be (Ficetola et al. 2018b).

In order to consider possible differences in the activity of the studied species, surveys were performed during the four seasons. The studied species are usually more active at the end of the cold season (Bale and Hayward 2010, Lunghi et al. 2018b, Lunghi et al. 2018c), thus spring surveys were the most numerous. Increasing the number of surveys within a short period allows the increase of robustness of analyses related to detection probability and population size estimation (Banks-Leite et al. 2014, Ficetola et al. 2018b).

The target species are not obligate cave species and they likely show day/night changes in their activity pattern (Mammola and Isaia 2018); our methodology allowed us to avoid this potential bias. Species abundances obtained from each seasonal pair of survey can be merged to obtain an average seasonal species abundance; futhermore, the survey with the highest number of observations can be set as the maximum observed seasonal abundance.

The fine-scale standardised methodology described here allows us to characterise the multiple subterranean microhabitats (Campbell Grant et al. 2007) and collect detailed information on species abundance (Lunghi et al. 2015, Lunghi et al. 2017, Lunghi et al. 2018a, Lunghi 2018).

This methodology, based on just observations, is appropriate for monitoring protected species (Ficetola et al. 2016, Lunghi et al. 2016, Ficetola et al. 2017), providing a practical tool compatible with restrictions imposed by local and international law (Stoch and Genovesi 2016).

## Geographic coverage

### Description

The Monte Albo is listed as a Site of Community Importance (SCI) by European law (European Commission Habitats Directive 92/43/EEC), as it represents an important biodiversity hotspot including several endangered species (AA. VV. 2006, Mulargia et al. 2018); nonetheless, it also represents the whole distribution range of the Monte Albo cave salamander *Hydromantes flavus* (Lanza et al. 2006).

### Coordinates

40.4379 and 40.5701 Latitude; 9.5110 and 9.6815 Longitude.

## Taxonomic coverage

### Description

The presence and abundance of the following five cave predators were recorded: The Monte Albo cave salamander *Hydromantes flavus*, the spiders *Meta bourneti*, *Metellina merianae* and *Tegenaria* sp., the land snail *Oxychilus oppressus* (Fig. 2). Species identification of *Tegenaria* in the field is not possible without handling animals (Bolzern et al. 2013); to avoid disturbance, all records refer to the genus. The monitored species show different life traits (Lunghi et al. 2017, Lunghi 2018) and are at the top of the cave food chain, providing a top-down control to other cave-dwelling invertebrates; however, interactions between these predator species are also possible (Pastorelli and Laghi 2006, Manenti et al. 2016, Lunghi et al. 2018c). The Monte Albo cave salamander is a local endemism that deserves particular protection, as its conservation status is considered Vulnerable by the IUCN Red List (Lanza et al. 2006, Lecis et al. 2009, Rondinini et al. 2013).

## Temporal coverage

**Data range:** 2015-10-14 – 2016-6-27.

## Usage rights

### Use license

Creative Commons Public Domain Waiver (CC-Zero)

## Data resources

### Data package title

Dataset_Monte_Albo

### Number of data sets

1

### Data set 1.

#### Data set name

data_monte_albo

#### Data format

Semi-colon delimited text file (.csv)

#### Number of columns

39

#### Description

Detailed information on cave features and abundance of five predator species (Suppl. material 1).

**Data set 1.** 

Column label	Column description
country	The name of the country in which the sampling was performed
region	The name of the region in which the sampling was performed
county	The name of the county in which the sampling was performed
locationID	The unique name of the surveyed location
eventDate	The date in which the survey was performed
eventSeason	The season in which the survey was performed
decimalLatitude	Coordinates of the latitute in WGS84 decimal degrees (N)
decimalLongitude	Coordinates of the longitude in WGS84 decimal degrees (E)
ElevationInMetres	Elevation (m a.s.l.) of the surveyed site
entrance_heightValue	The maximum height of the cave entrance (m)
entrance_widthValue	The maximum width of the cave entrance (m)
external_temperatureValue	The outdoor air temperature (°C) measured at 5-10 m from the cave entrance
external_humidityValue	The outdoor air humidity (%) measured at 5-10 m from the cave entrance
branchID	The number of the cave branch
sector_depthValue	The linear distance (m) of the sector from the cave entrance
sector_heightValue	The maximum height (m) of the cave sector
sector_widthValue	The maximum width (m) of the cave sector
sector_wall_irregularityValue	The average wall irregularity (m) of the cave sector. This value tends to 1 when cave walls show low irregularity, while it gets smaller when the wall irregularity increases
sector_temperatureValue	The seasonal average air temperature (°C) of the cave sector
sector_humidityValue	The seasonal average air humidity (%) of the cave sector
sector_max_illuminanceValue	The seasonal average maximum illuminance (lx) of the cave sector
sector_min_illuminanceValue	The seasonal average minimum illuminance (lx) of the cave sector
Hydromantes_flavusQuantityType	The typology of data recorded for *Hydromantes flavus* Stefani, 1969 (Urodela: Plethodontidae): individual
Hydromantes_flavusQuantity	Number of observed *Hydromantes flavus*
Meta_bournetiQuantityType	The typology of data recorded for *Meta bourneti* (Roberts 1995) (Araneae: Tetragnathidae): individual
Meta_bournetiQuantity	Number of observed *Meta bourneti*
Meta_bourneti_eggQuantityType	The typology of data recorded for *Meta bourneti* egg sacks: cocoons
Meta_bourneti_eggQuantity	Number of observed *Meta bourneti* egg sacks
TegenariaQuantityType	The typology of data recorded for *Tegenaria* Latreille, 1804 (Araneae: Agelenidae): individual
TegenariaQuantity	Number of observed *Tegenaria*
Tegenaria_eggQuantityType	The typology of data recorded for *Tegenaria* egg sacks: cocoons
Tegenaria_eggQuantity	Number of observed *Tegenaria* egg sacks
Metellina_merianaeQuantityType	The typology of data recorded for *Metellina merianae* (Scopoli, 1763) (Araneae: Tetragnathidae): individual
Metellina_merianaeQuantity	Number of observed *Metellina merianae*
Metellina_merianae_eggQuantityType	The typology of data recorded for *Metellina merianae* egg sacks: cocoons
Metellina_merianae_eggQuantity	Number of observed *Metellina merianae* egg sacks
Oxychilus_oppressusQuantityType	The typology of data recorded for *Oxychilus oppressus* (Shuttleworth, 1877) (Gastropoda: Oxychilidae): individual
Oxychilus_oppressusQuantity	Number of observed *Oxychilus oppressus*
recordedBy	The Name and Surname of person recording the data

## Supplementary Material

B431F286-7F9D-513B-A2F6-96BD581CCD87 10.3897/BDJ.8.e48623.suppl1 Supplementary material 1 Dataset_Monte_Albo Data type: Dataset
Brief description: Detailed information on morphology and microclimate of the surveyed caves in the Monte Albo massif and number of observed individuals belonging to five predator species exploiting these caves. For spiders, the number of the observed cocoons is also reported. NA means that the data is not available.
File: oo_375031.csv https://binary.pensoft.net/file/375031 Enrico Lunghi, Claudia Corti, Manuela Mulargia, Yahui Zhao, Raoul Manenti, Gentile Francesco Ficetola, Michael Veith

## Figures and Tables

**Figure 2a.**
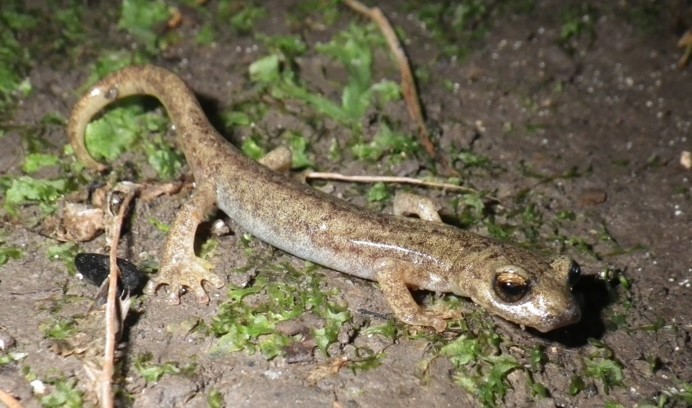
*Hydromantes flavus* Stefani, 1969 (Urodela: Plethodontidae)

**Figure 2b.**
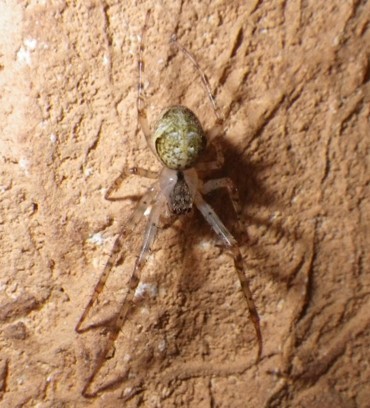
*Metellina merianae* (Scopoli, 1763) (Araneae: Tetragnathidae)

**Figure 2c.**
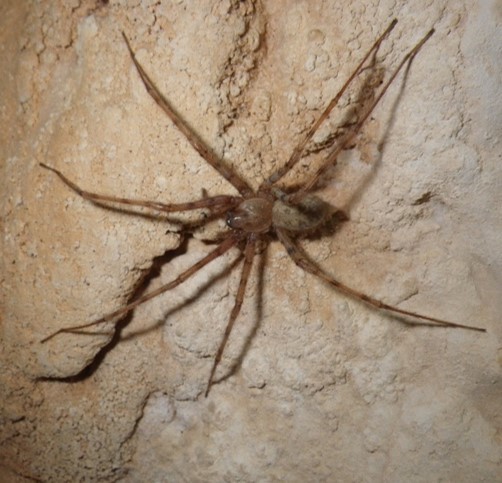
*Tegenaria* sp. Latreille, 1804 (Araneae: Agelenidae)

**Figure 2d.**
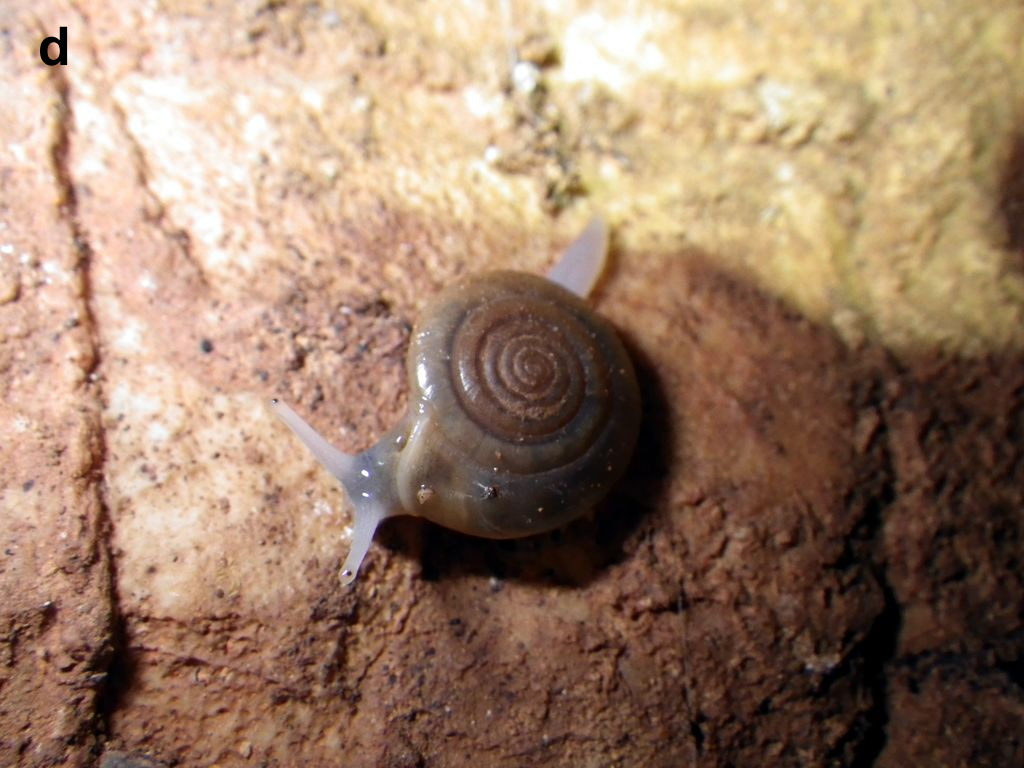
*Oxychilus oppressus* (Shuttleworth, 1877) (Gastropoda: Oxychilidae)

**Figure 2e.**
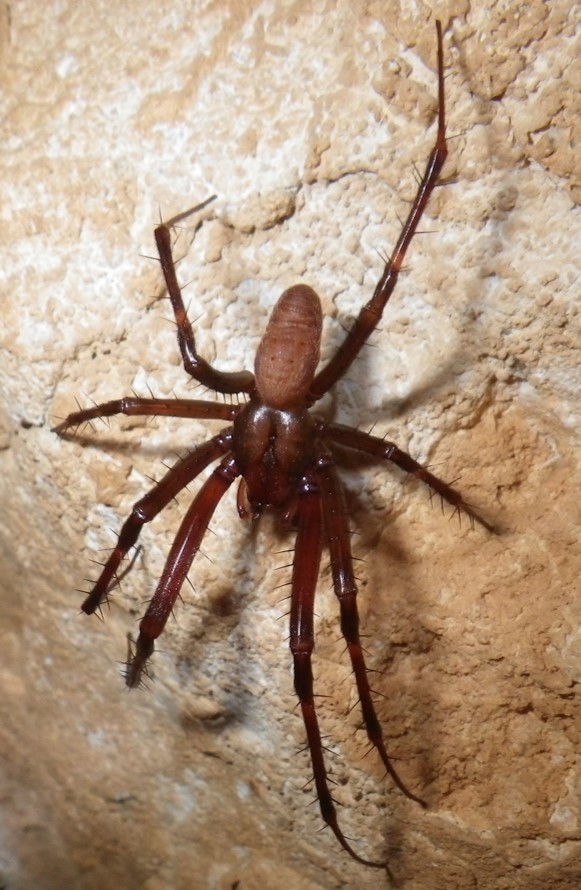
*Meta bourneti* (Roberts 1995) (Araneae: Tetragnathidae)
